# The Influence of Satellite Configuration and Fault Duration Time on the Performance of Fault Detection in GNSS/INS Integration

**DOI:** 10.3390/s19092147

**Published:** 2019-05-09

**Authors:** Chuang Zhang, Xiubin Zhao, Chunlei Pang, Liang Zhang, Bo Feng

**Affiliations:** 1 Information and Navigation College, Air Force Engineering University, Xi’an 710077, China; zhangchuanglw@163.com (C.Z.); zhaoxiubin926@163.com (X.Z.); zhangliang_nudt@nudt.edu.cn (L.Z.); 2 Equipment Management and Unmanned Aerial Vehicle Engineering College, Air Force Engineering University, Xi’an 710051, China; fengbo876@163.com

**Keywords:** GNSS/INS integration, fault detection, chi-square test, satellite configuration, fault duration time

## Abstract

For the integration of global navigation satellite system (GNSS) and inertial navigation system (INS), real-time and accurate fault detection is essential to enhance the reliability and precision of the system. Among the existing methods, the residual chi-square detection is still widely used due to its good real-time performance and sensibility of fault detection. However, further investigation on the performance of fault detection for different observational conditions and fault models is still required. In this paper, the principle of chi-square detection based on the predicted residual and least-squares residual is analyzed and the equivalence between them is deduced. Then, choosing the chi-square detection based on the predicted residual as the research object, the influence of satellite configuration and fault duration time on the performance of fault detection is analyzed in theory. The influence of satellite configuration is analyzed from the number and geometry of visible satellites. Several numerical simulations are conducted to verify the theoretical analysis. The results show that, for a single-epoch fault, the location of faulty measurement and the geometry have little effect on the performance of fault detection, while the number of visible satellites has greater influence on the fault detection performance than the geometry. For a continuous fault, the fault detection performance will decrease with the increase of fault duration time when the value of the fault is near the minimal detectable bias (MDB), and faults occurring on different satellite’s measurement will result in different detection results.

## 1. Introduction

Due to the good complementary characteristics, the integration of the inertial navigation system (INS) and the global navigation satellite system (GNSS) can achieve superior performance to either of them operating alone [1,2], and is widely applied on unmanned aerial vehicles (UAVs). The GNSS/INS integration can obtain the optimal estimates of the navigation parameters by using a Kalman filter with correct state and observation models. As a self-contained system, INS is immune to jamming and interference [3,4]. Hence, it is usually considered as the common reference system and assumed absolutely reliable. However, the GNSS measurements are easy to be interfered and may contain faults. In this case, the integration can’t offer reliable and accurate navigation information due to the fault observations [5]. In general, the navigation sensors faults can be classified into two types in the time domain: the single-epoch fault and continuous fault. Among these two types, the continuous fault has a greater influence on the filtering precision due to the long duration time. If the fault is not timely diagnosed, the whole navigation will be polluted and the navigation precision will degrade [6]. Therefore, it is necessary to carry out the real-time fault detection and isolation to ensure the reliability and precision of the integrated navigation system [7].

Until now, many mature fault detection methods have been proposed, which can be classified into three categories, i.e., hardware redundancy methods, analytical redundancy methods, and nonanalytical redundancy methods [6,8]. The hardware redundancy configuration usually exceeds the minimum necessary and increase the cost of the navigation equipment [9,10]. The nonanalytical redundancy methods essentially are data-driven methods based on machine learning [6]. These methods have good performance in detection and identification for nonlinear systems and uncertainty of system models. The representative methods mainly include artificial neural network (ANN) [9,11,12], support vector machine (SVM) [13], and Gaussian process regression (GPR) [14]. The major challenge of this kind of method is how to build an appropriate regressive model depending on the input/output data. Another drawback that needs to be overcome is that these methods usually involve a large amount of calculation and are hard to meet the requirement of real-time performance.

The chi-square detection method, which belongs to the analytical redundancy category and has less calculation, is the classical method and still widely used [15,16,17]. Brumback and Srinath proposed a chi-square test based on the difference between the two state estimates for fault detection in Kalman filters [18]. Using recursive filtering and the residual chi-square test, an integrity and quality control procedure called detection, identification, and adaptation (DIA) was investigated by Teunissen [19]. Compared to the state chi-square detection method, the residual chi-square detection method can detect the abrupt fault in time with a small amount of computation, but it does not work well in gradual fault detection [10]. In order to improve the performance of chi-square fault detection method for gradual fault, many improved methods had been proposed. Based on the chi-square detection, the autonomous integrity monitoring by extrapolation (AIME) method in which the measurements used are not limited to a single epoch was proposed [20]. Solution separation is another fault detection method which uses the difference between the main filter solution and the subfilter solution to determine the test statistic. The representative methods are multiple solution separation (MSS) [21,22] and normalized solution separation (NSS) [7,23]. The performance of MSS and AIME for gradual fault were tested and the analysis revealed that both methods had advantages and disadvantages [24]. MSS guarantees satisfactory detection performance theoretically, but it has heavy calculation burden due to the design of multiple filters. AIME can achieve higher availability; however, there is no good way to confirm the detection performance based on theory. A new rate detector algorithm based on AIME has been developed [4] and the test results show that the rate detector algorithm has better detection performance than AIME for gradual faults. The approach for detecting the gradual fault based on least squares support vector machine (LS-SVM) and AIME was proposed in [25]. Based on the replaced innovation obtained from the LS-SVM, the test statistics can follow fault amplitudes more accurately.

The above methods are mainly based on the predicted residual of Kalman filter. In geodetic surveying, the commonly used method is based on the least-squares residual (estimated residual) [26]. In order to apply the related methods of GNSS receiver autonomous integrity monitoring (RAIM) into GNSS/INS integration, Hewitson proposed an extended RAIM (eRAIM) through adopting least-squares principles for the state estimation in a Kalman filter [27]. This method formed the test statistic basing on the least-squares residual obtained by integrating the measurements with one-step prediction of the state parameters, and it can detect faults in the dynamic model and isolate them from the measurement model. The relation between the predicted residual vector-based chi-square test and the estimated residual vector-based chi-square test is not given. In this contribution, we analyze the principle of these two methods and give the equivalence proof.

Most previous studies didn’t consider the influence of satellite configuration on the performance of fault detection, and this influence is mainly reflected in the geometry and the number of visible satellites. When the number of satellites is given, the geometry of visible satellites will have an influence on the filtering precision of the integration. Zaminpardaz et al. [28] analyzed how geometry changes in the measurement setup affect the testing procedure of the DIA method. However, they didn’t analyze the influence of the number of visible satellites on the performance of fault detection. That analysis was mainly focused on GNSS and the related analysis for GNSS/INS integration was not discussed. Wang et al. [29] conducted fault separability analysis for multiple faults in GNSS/INS integration. This analysis investigated the impact of the number of visible satellites, satellite geometry, and the number of system state models on the correlation coefficients between fault detection test statistics. This work mainly analyzed the influence on the fault separability, but the analysis of the influence on fault detection is not researched. Hence, there is a need to analyze how changes in the geometry and number of visible satellites affects the performance of fault detection in GNSS/INS integration.

The residual chi-square method is a global test method which evaluates the quality of measurement in system level [30]. For tightly-coupled GNSS/INS integration, identifying and isolating the faulty measurement correctly is another key issue of fault detection. Usually, a local test is conducted to identify the outlier after the global test is accomplished. The commonly used identification method is the data snooping based on Baarda’s w-test [31]. If the global test is rejected, the measurement fault can be identified by the local test [32]. After that, the filter measurement update will be done by using the normal measurements. However, this scheme ignores the influence of the fault duration time on the performance of fault detection. Differing from the detection method based on the least-squares residual in geodetic surveying, the Kalman filter is a regressive process, and the previous detection and filtering results will have a great influence on the later detection and state estimation precision. For a single-epoch fault, due to the short duration time, isolating the faulty measurement will hardly bring bad influence on the filtering precision and the fault detection performance. However, for a continuous fault, isolating the faulty measurement may result in the degradation of the filter’s precision and the sensitivity of fault detection, and for different measurement conditions and fault duration time, the degradation may be variant. Nevertheless, most of the previous studies mainly aimed at reducing the time delay of gradual fault detection, the influence of measurement conditions and fault duration time on fault detection after fault identification and isolation was seldom taken into account, especially for abrupt faults. Among the two types of fault, the amplitude of a gradual fault increases with fault duration time, while an abrupt fault can be regarded as a constant fault error during a period of time. Hence, the satellite configuration and fault duration time may have greater influence on the abrupt fault detection than the gradual fault detection after fault identification and isolation. Therefore, there is a need to analyze the influence of measurement conditions and fault duration time on the performance of abrupt fault detection.

In this contribution, to have an overall understanding of the performance of the residual chi-square detection method and deepen the application of it in GNSS/INS integration, we analyze the principle of two residual chi-square detection methods and give the equivalence deduction of them in theory. Then, the influence of the satellite configuration and fault duration time on the performance of fault detection for abrupt fault is analyzed. In addition, several numerical simulation tests are conducted to verify the conclusion of the theoretical analysis.

The rest of this paper is organized as follows. The GNSS/INS integration model is given in Section 2. The principle of chi-square detection based on the predicted residual and least-squares residual is analyzed and the equivalence between them is deducted in Section 3. Section 4 analyzes how the satellite configuration and fault duration time affect the performance of fault detection. Simulation results and analysis are shown in Section 5. Finally, the conclusions are drawn in Section 6.

## 2. GNSS/INS Integration Model

### 2.1. GNSS/INS Integration State Model

For tightly-coupled GNSS/INS integration, the system state model consists of the error state equations of both INS and GNSS. The state vector is usually chosen as

(1) $$X={\left[\phi_{E},\phi_{N},\phi_{U},\delta v_{E},\delta v_{N},\delta v_{U},\delta L,\delta \lambda ,\delta h,\varepsilon_{x},\varepsilon_{y},\varepsilon_{z},\nabla_{x},\nabla_{y},\nabla_{z},\delta t_{u},\delta t_{ru}\right]}^{T}$$

where $\phi_{E}$, $\phi_{N}$, $\phi_{U}$ are the misalignment angles, $\delta v_{E}$, $\delta v_{N}$, $\delta v_{U}$ are the east, north, and upward velocity errors, respectively. δL, δλ, δh denote the latitude, longitude, and height errors, $\varepsilon_{x}$, $\varepsilon_{y}$, $\varepsilon_{z}$ and $\nabla_{x}$, $\nabla_{y}$, $\nabla_{z}$ represent the gyro biases and accelerometer biases. $\delta t_{u}$, $\delta t_{ru}$ are the range bias and range drift related to the receiver clock.

### 2.2. GNSS/INS Integration Measurement Model

In the measurement model, the observation vector consists of the pseudorange differences between INS and GNSS. The system measurement equation can be written as

(2) Z=HX+V

where Z denotes the measurement vector, H denotes the measurement matrix, and V denotes the measurement noise vector. When the number of visible satellites is n, it can be obtained

(3) $$Z=\left[\begin{array}{c} \delta \rho_{1}\\ \delta \rho_{2}\\ \cdots \\ \delta \rho_{n} \end{array}\right]=\left[\begin{array}{c} \rho_{I}^{1}\\ \rho_{I}^{2}\\ \cdots \\ \rho_{I}^{n} \end{array}\right]-\left[\begin{array}{c} \rho_{G}^{1}\\ \rho_{G}^{2}\\ \cdots \\ \rho_{G}^{n} \end{array}\right],H=\left[\begin{array}{cccc} 0_{n\times 6} & H_{\rho 1} & 0_{n\times 6} & H_{\rho 2} \end{array}\right]$$

where $\rho_{I}^{i}$, $\rho_{G}^{i}$(i=1,2,⋯,n) denote the pseudorange of INS and GNSS, respectively. $H_{\rho 1}$ and $H_{\rho 2}$ are matrices that denote the relationship between measurements and state vector [25].

## 3. Fault Detection and Isolation Based on Chi-Square Test

### 3.1. Fault Detection Based on Chi-Square Test

#### 3.1.1. Principle of Chi-Square Detection Based on Predicted Residual

The linear discrete time varying system model can be described as

(4) $$\{\begin{array}{l} X_{k}=\Phi_{k,k-1}X_{k-1}+\Gamma_{k-1}W_{k-1}\\ Z_{k}=H_{k}X_{k}+V_{k} \end{array}$$

where $X_{k}$ is the state vector, $\Phi_{k,k-1}$ is the transition matrix, $\Gamma_{k-1}$ is the coefficient matrix, $Z_{k}$ represents the measurement vector, $H_{k}$ is the measurement model matrix. $W_{k}$ is the process noise which is commonly assumed as a zero-mean Gaussian white noise with covariance matrix $Q_{k}$, and $V_{k}$ is the measurement noise which is commonly assumed as a zero-mean Gaussian white noise with covariance matrix $R_{k}$. $W_{k}$ and $V_{k}$ are independent.

The one step prediction of the state in Kalman filter is

(5) $${\bar{X}}_{k,k-1}=\Phi_{k,k-1}{\hat{X}}_{k-1}$$

The predicted residual vector is

(6) $${\bar{v}}_{k}=Z_{k}-H_{k}{\bar{X}}_{k,k-1}$$

The covariance of the predicted residual vector is

(7) $$P_{\bar{v}k}=H_{k}P_{k,k-1}H_{k}^{T}+R_{k}$$

where $P_{k,k-1}$ is the covariance matrix of state prediction ${\bar{X}}_{k,k-1}$.

When no fault occurs, the residual vector is white noise and its mean is 0. If the measurement vector contains a fault, the statistical characteristics of the residual will change, and its mean is no longer equal to 0. Define two hypotheses, the null hypothesis $H_{0}$ and the alternative hypothesis $H_{1}$.

$H_{0}$ denotes no fault and can be expressed as

(8) $$H_{0}:{\bar{v}}_{k}∼N\left(0,P_{\bar{v}k}\right)$$

$H_{1}$ denotes the existence of a fault and can be given by

(9) $$H_{1}:{\bar{v}}_{k}∼N\left(\mu ,P_{\bar{v}k}\right)$$

where μ is the mean of ${\bar{v}}_{k}$.

Then, the fault detection function is

(10) $${\bar{\Lambda }}_{k}={\bar{v}}_{k}^{T}P_{\bar{v}k}^{-1}{\bar{v}}_{k}$$

where ${\bar{\Lambda }}_{k}$ obeys the $\chi^{2}$ distribution and its degree of freedom is the dimension of observations vector $Z_{k}$. Under the null hypothesis, ${\bar{\Lambda }}_{k}∼\chi^{2}\left(0,n\right)$, while under the alternative hypothesis, ${\bar{\Lambda }}_{k}∼\chi^{2}\left(\sigma ,n\right)$, where σ is the noncentrality parameter and can be obtained as:

(11) $$\sigma =\mu^{T}P_{\bar{v}k}^{-1}\mu$$

When the false alarm rate is defined as α, according to the Neyman–Pearson criterion, the threshold $T_{d}$ can be worked out through solving the equation $P\left\{{\bar{\Lambda }}_{k}>T_{d}\right\}=\alpha$ [10]. The fault detection criteria is:

(12) $$\{\begin{array}{cc} {\bar{\Lambda }}_{k}>T_{d} & \mathrm{fault}\;\mathrm{occurs}\\ {\bar{\Lambda }}_{k}\leq T_{d} & \mathrm{no}\;\mathrm{fault}\;\mathrm{occurs} \end{array}$$

#### 3.1.2. Principle of Chi-square Detection Based on Least-Squares Residual

For the linear discrete system (4), the state estimation of Kalman filter and its covariance matrix can be expressed as

(13) $$\{\begin{array}{l} {\hat{X}}_{k}={\bar{X}}_{k,k-1}+K_{k}\left(Z_{k}-H_{k}{\bar{X}}_{k,k-1}\right)\\ P_{k}=\left(I-K_{k}H_{k}\right)P_{k,k-1} \end{array}$$

Then, the least-squares residual vector can be expressed as

(14) $$v_{k}=Z_{k}-H_{k}{\hat{X}}_{k}$$

The corresponding covariance matrix is

(15) $$P_{vk}=R_{k}-H_{k}P_{k}H_{k}^{T}$$

In the same way, the fault detection function can be expressed as

(16) $$\Lambda_{k}=v_{k}^{T}P_{vk}^{-1}v_{k}$$

where $\Lambda_{k}$ obeys the $\chi^{2}$ distribution and its degree of freedom is the dimension of measurement vector $Z_{k}$. The fault detection criteria is the same as Equation (12).

#### 3.1.3. Equivalence Analysis of the Two Methods

According to the Kalman filter equations and matrix theory, the relation of least-squares residual vector and the predicted residual vector can be expressed as

(17) $$v_{k}=\left(I-H_{k}K_{k}\right){\bar{v}}_{k}$$

where $K_{k}=P_{k,k-1}H_{k}^{T}P_{\bar{v}k}^{-1}$ is the filter gain matrix. Hence, the above equation can be written as

(18) $${\bar{v}}_{k}=P_{\bar{v}k}R_{k}^{-1}v_{k}$$

Combining Equations (10) and (18), we can get

(19) $$\begin{array}{cc} {\bar{\Lambda }}_{k} & ={\bar{v}}_{k}^{T}P_{\bar{v}k}^{-1}{\bar{v}}_{k}\\ & ={\left(P_{\bar{v}k}R_{k}^{-1}v_{k}\right)}^{T}P_{\bar{v}k}^{-1}P_{\bar{v}k}R_{k}^{-1}v_{k}\\ & =v_{k}^{T}R_{k}^{-1}P_{\bar{v}k}R_{k}^{-1}v_{k} \end{array}$$

According to Equation (15), it can be obtained that

(20) $$\begin{array}{cc} P_{vk}^{-1} & ={\left(R_{k}-H_{k}P_{k}H_{k}^{T}\right)}^{-1}\\ & =R_{k}^{-1}+R_{k}^{-1}H_{k}{\left(P_{k}^{-1}-H_{k}^{T}R_{k}^{-1}H_{k}\right)}^{-1}H_{k}^{T}R_{k}^{-1}\\ & =R_{k}^{-1}+R_{k}^{-1}H_{k}P_{k,k-1}^{-1}H_{k}^{T}R_{k}^{-1}\\ & =R_{k}^{-1}\left(R_{k}+H_{k}P_{k,k-1}^{-1}H_{k}^{T}\right)R_{k}^{-1}\\ & =R_{k}^{-1}P_{\bar{v}k}R_{k}^{-1} \end{array}$$

Therefore, the fault detection function $\Lambda_{k}$ can be written as

(21) $$\begin{array}{cc} \Lambda_{k} & =v_{k}^{T}P_{vk}^{-1}v_{k}\\ & =v_{k}^{T}R_{k}^{-1}P_{\bar{v}k}R_{k}^{-1}v_{k} \end{array}$$

Combining Equations (19) and (21), we can get

(22) $${\bar{\Lambda }}_{k}=\Lambda_{k}$$

Equation (22) indicates that the fault detection function $\Lambda_{k}$ based on the least-squares residual and the fault detection function ${\bar{\Lambda }}_{k}$ based on the predicted residual are equivalent. The difference between them is that the calculation of ${\bar{\Lambda }}_{k}$ is done before the Kalman filter measurement update, while $\Lambda_{k}$ can just be calculated after the measurement update is finished. Hence, the fault detection based on the predicted residual is chosen as the research object, and the performance of it will be analyzed in the later parts.

### 3.2. Fault Isolation Based on Local Test

The residual chi-square detection method is a global detection method, it can’t identify the faulty measurement. To identify and isolate the faulty measurement, the local test method is introduced.

Assume the residual vector at time k is ${\bar{v}}_{k}$, then the fault detection function is given as [33]

(23) $$\lambda_{k}^{i}=\frac{c_{i}^{T}P_{\bar{v}k}^{-1}{\bar{v}}_{k}}{\sqrt{c_{i}^{T}P_{\bar{v}k}^{-1}c_{i}}},i=1,\cdots ,n$$

where $c_{i}$ is the unit vector with the ith element equal to one. $\lambda_{k}^{i}$ is the standardized residual.

The fault detection criteria is

(24) $$\{\begin{array}{cc} \left|\lambda_{k}^{i}\right|>N_{\alpha_{0}/2}\left(0,1\right) & \mathrm{fault}\;\mathrm{occurs}\\ \left|\lambda_{k}^{i}\right|\leq N_{\alpha_{0}/2}\left(0,1\right) & \mathrm{no}\;\mathrm{fault}\;\mathrm{occurs} \end{array}$$

Usually, the relationship between the probability of false alert for the ith pseudorange $\alpha_{0}$ and the global test probability of false alert α can be expressed as [34,35]

(25) $$\alpha_{0}=1-\sqrt[{n}]{1-\alpha }$$

Every dimensional measurement should be tested to identify the fault because the fault may occur on each measurement. After the fault identification, the faulty measurement is usually isolated, while the rest of the normal measurements are used to conduct the filter measurement update process.

## 4. Analysis of Influencing Factors of Fault Detection Performance

### 4.1. Performance Indicator of Residual Chi-Square Detection Method

To analyze the performance of residual chi-square detection method, the concept of the minimal detectable bias (MDB) is introduced. The MDB is defined as the minimal model error that can just be detected [36].

It is assumed that a fault occurs on the ith measurement, then the measurement model can be written as

(26) $$Z_{k}=H_{k}X_{k}+V_{k}+l_{i}\nabla$$

where ∇ is the fault error on the ith observation, and $l_{i}={\left(0,0,\cdots ,1,\cdots ,0\right)}^{T}$ is the unit vector with the ith element equal to one, when the residual vector is

(27) $${\bar{v}}_{k}=Z_{k}-H_{k}{\bar{X}}_{k,k-1}=v_{k}+l_{i}\nabla$$

which has the expectation

(28) $$E\left({\bar{v}}_{k}\right)=\mu =l_{i}\nabla$$

Then, the noncentrality parameter of fault detection function can be expressed as

(29) $$\sigma_{\nabla }=\mu^{T}P_{\bar{v}k}^{-1}\mu ={\left(l_{i}\nabla \right)}^{T}P_{\bar{v}k}^{-1}l_{i}\nabla =\nabla^{2}P_{\bar{v}k}^{-1}{}_{ii}$$

where $P_{\bar{v}k}^{-1}{}_{ii}$ is the ith diagonal element of covariance matrix $P_{\bar{v}k}^{-1}$.

The MDB for the ith diagonal observation can be obtained as

(30) $${\left|\nabla \right|}_{\min}=\sqrt{\sigma /P_{\bar{v}k}^{-1}{}_{ii}}$$

where σ is the noncentrality parameter, which is a function of α and the probability of missed detection β, and can be given by

(31) $$\sigma =\chi_{\alpha }^{2}\left(n\right)-\chi_{1-\beta }^{2}\left(n\right)$$

The larger the value of fault, the easier the fault can be detected. When the value of fault is smaller than MDB, it is difficult to detect the fault accurately and the probability of missed detection will increase.

### 4.2. The Influence of Satellite Configuration on the Performance of Fault Detection for a Single-Epoch Fault

From the above analysis, it can be seen that the MDB is related to the noncentrality parameter σ and covariance $P_{\bar{v}k}^{-1}{}_{ii}$. For certain probabilities of false alarm and missed detection, when the number of visible satellites is given, the noncentrality parameter σ is constant, the MDB is only related to $P_{\bar{v}k}^{-1}{}_{ii}$, and the relation between them is negative. Hence, define $1/P_{\bar{v}k}^{-1}{}_{ii}$ as the dilution of minimal detectable bias (DOMDB), and the larger the DOMDB, the larger the MDB.

According to Equation (7), it can be obtained that

(32) $$\begin{array}{l} P_{\bar{v}k}^{-1}={\left(H_{k}P_{k,k-1}H_{k}^{T}+R_{k}\right)}^{-1}\\ =R_{k}^{-1}-R_{k}^{-1}H_{k}{\left(P_{k,k-1}^{-1}+H_{k}^{T}R_{k}^{-1}H_{k}\right)}^{-1}H_{k}^{T}R_{k}^{-1} \end{array}$$

$P_{\bar{v}k}^{-1}{}_{ii}$ is the ith diagonal element of covariance matrix $P_{vk}^{-1}$, and can be expressed as

(33) $$\begin{array}{l} P_{\bar{v}k}^{-1}{}_{ii}=R_{k}^{-1}{}_{ii}-H_{k}^{i}{\left(P_{k,k-1}^{-1}+H_{k}^{T}R_{k}^{-1}H_{k}\right)}^{-1}{\left(H_{k}^{i}\right)}^{T}{\left(R_{k}^{-1}{}_{ii}\right)}^{2}\\ =R_{k}^{-1}{}_{ii}-H_{k}^{i}P_{k}{\left(H_{k}^{i}\right)}^{T}{\left(R_{k}^{-1}{}_{ii}\right)}^{2}\\ =\left[R_{k}^{ii}-H_{k}^{i}P_{k}{\left(H_{k}^{i}\right)}^{T}\right]{\left(R_{k}^{-1}{}_{ii}\right)}^{2} \end{array}$$

where $R_{k}^{-1}{}_{ii}$ is the ith diagonal element of covariance matrix $R_{k}^{-1}$, $R_{k}^{ii}$ is the ith diagonal element of covariance matrix $R_{k}$, $H_{k}^{i}$ is the ith row of $H_{k}$. From the above equation, it can be seen that $P_{\bar{v}k}^{-1}{}_{ii}$ is related to the accuracy of GNSS observation, the state estimation covariance matrix $P_{k}$ and the corresponding measurement matrix $H_{k}^{i}$. And $P_{\bar{v}k}^{-1}{}_{ii}$ is negatively related to $P_{k}$ and $R_{k}$, while $P_{k}$ is negatively related to process noise covariance matrix $Q_{k}$. In other words, the worse the accuracy of IMU and GNSS measurement, the smaller the value of $P_{\bar{v}k}^{-1}{}_{ii}$, and the lower the sensibility of fault detection. For a determined integration, the accuracy of IMU and GNSS measurement is constant, so $P_{\bar{v}k}^{-1}{}_{ii}$ is only related to the measurement matrix $H_{k}^{i}$ and state estimation covariance matrix $P_{k}$, while $P_{k}$ depends on the measurement matrix $H_{k}$ to a great extent. The different geometry of satellites will lead to the difference of measurement matrix $H_{k}$, which will result in the different precision of $P_{k}$.

When the geometry is good, after the Kalman filter is converged, $P_{k}$ tends to be a stable value close to 0, although the difference of measurement matrix $H_{k}$ results in the different precision of $P_{k}$, the difference of precision is very small, which means that $P_{\bar{v}k}^{-1}{}_{ii}$ mainly depends on $R_{k}^{ii}$. When the geometry is poor, the filtering error will get larger, for the same satellite, the value of $P_{\bar{v}k}^{-1}{}_{ii}$ will get smaller. However, compared to the value of $R_{k}^{ii}$, the change of the value of $H_{k}^{i}P_{k}{\left(H_{k}^{i}\right)}^{T}$ caused by the increase of filtering error is very small. In other words, the increase of filtering error will not cause a very noticeable change in the value of $P_{\bar{v}k}^{-1}{}_{ii}$, which has two meanings. One is that although different geometry of satellites will result in the different precision of $P_{k}$, this will have a very small influence on the covariance $P_{\bar{v}k}^{-1}{}_{ii}$. In other words, the geometry of the satellites will minorly affect the performance of fault detection. The second meaning is that for a given geometry, although each of the satellites has different elevation and azimuth, the $P_{\bar{v}k}^{-1}{}_{ii}$ of them are similar. That is to say, for single-epoch fault detection, no matter which satellite’s measurement has the fault error, the difference of fault detection results will be very small. This conclusion is quite different from that in GNSS RAIM. Of course, the premise of the above conclusion is that the filtering error is not very large and the fault detection method is available. Just like the geometry would affect the availability of RAIM, if the geometry is so poor that the filtering error is too large, the availability of the fault detection method will be challenged.

When the number of satellites varies, the noncentrality parameter σ and the threshold $T_{d}$ will change too, and they will increase with the growth of the number. Besides, the growth of the visible satellites’ number will also change the measurement matrix, which will affect the state estimation precision directly. Hence, these two factors will affect the performance of fault detection.

### 4.3. The Influence of Fault Duration Time and Geometry on the Performance of Fault Detection for a Continuous Fault

The single-epoch fault has little impact on the filtering precision, whereas a continuous fault occurring during a period of time has a greater effect on the filtering precision, which may cause the filtering precision get worse, and even result in divergence. There is a need to analyze the influence of the fault duration time on the filtering precision and fault detection performance.

The traditional fault detection and isolation (FDI) method conducts the measurement update of Kalman filter by using the normal measurements after the fault isolation. For the convenience of analysis, assume that fault occurs on the ith dimension of the measurement vector, then the new measurement matrix ${\tilde{H}}_{k}$ after the fault isolation can be written as

(34) $${\tilde{H}}_{k}=\left[H_{k}^{1};\cdots ;H_{k}^{i-1};H_{k}^{i+1};\cdots ;H_{k}^{n}\right]$$

where $H_{k}^{i}$ is the ith row element of $H_{k}$. Then, the new state estimation covariance matrix ${\tilde{P}}_{ki}$ can be written as ${\tilde{P}}_{ki}={\left({\tilde{H}}_{k}^{T}{\tilde{R}}_{k}^{-1}{\tilde{H}}_{k}+P_{k,k-1}^{-1}\right)}^{-1}$, where ${\tilde{R}}_{k}$ is the noise covariance matrix of the remainder measurements. According to the matrix theory, the relation between $P_{k}$ and ${\tilde{P}}_{ki}$ can be expressed as

(35) $$\begin{array}{l} P_{k}={\left({\tilde{H}}_{k}^{T}{\tilde{R}}_{k}^{-1}{\tilde{H}}_{k}+P_{k,k-1}^{-1}+{\left(H_{k}^{i}\right)}^{T}\delta^{-2}H_{k}^{i}\right)}^{-1}\\ ={\left({\tilde{P}}_{ki}^{-1}+{\left(H_{k}^{i}\right)}^{T}\delta^{-2}H_{k}^{i}\right)}^{-1}\\ ={\tilde{P}}_{ki}-{\tilde{P}}_{ki}{\left(H_{k}^{i}\right)}^{T}{\left(\delta^{2}+H_{k}^{i}{\tilde{P}}_{ki}{\left(H_{k}^{i}\right)}^{T}\right)}^{-1}H_{k}^{i}{\tilde{P}}_{ki}\\ ={\tilde{P}}_{ki}-\Delta P_{k} \end{array}$$

where $\delta^{2}$ is the variance of the measurement error. From the above equation, it can be proved that the following equation is established

(36) $$P_{k}^{jj}\leq {\tilde{P}}_{ki}^{jj}$$

where $P_{k}^{jj}$ and ${\tilde{P}}_{ki}^{jj}$ are the jth diagonal element of covariance matrix $P_{k}$ and ${\tilde{P}}_{ki}$, respectively.

Equation (36) indicates that the state estimation error after fault isolation will be larger than that of a filter without faulty measurement. The Kalman filter is a continuous recursion process, thus the current filter results will have an influence on the next filter process. If a fault error still exists and occurs on the ith dimension of the measurement vector at time k+1, after the fault detection and isolation, the state estimation covariance matrix of the Kalman filter can be written as

(37) $${\tilde{P}}_{(k+1)i}={\left({\tilde{H}}_{k+1}^{T}{\tilde{R}}_{k+1}^{-1}{\tilde{H}}_{k+1}+{\tilde{P}}_{k+1,k}^{-1}\right)}^{-1}$$

where ${\tilde{P}}_{k+1,k}=\Phi_{k+1,k}{\tilde{P}}_{ki}\Phi_{k+1,k}^{T}+\Gamma_{k+1,k}Q_{k}\Gamma_{k+1,k}^{T}$ is the covariance matrix of the predicted state at time k+1. The corresponding covariance matrix of state estimation without fault can be expressed as

(38) $$P_{k+1}={\left(H_{k+1}^{T}R_{k+1}^{-1}H_{k+1}+P_{k+1,k}^{-1}\right)}^{-1}$$

where $P_{k+1,k}=\Phi_{k+1,k}P_{k}\Phi_{k+1,k}^{T}+\Gamma_{k+1,k}Q_{k}\Gamma_{k+1,k}^{T}$. According to Equation (35), the relation between $P_{k+1,k}$ and ${\tilde{P}}_{k+1,k}$ can be given as

(39) $$P_{k+1,k}={\tilde{P}}_{k+1,k}-\Phi_{k+1,k}\Delta P_{k}\Phi_{k+1,k}^{T}$$

Inverting both sides of Equation (39), we can get

(40) $$\begin{array}{l} P_{k+1,k}^{-1}={\left({\tilde{P}}_{k+1,k}-\Phi_{k+1,k}\Delta P_{k}\Phi_{k+1,k}^{T}\right)}^{-1}\\ ={\tilde{P}}_{k+1,k}^{-1}+{\tilde{P}}_{k+1,k}^{-1}\Phi_{k+1,k}\\ {\left[{\left(\Delta P_{k}\right)}^{-1}+\Phi_{k+1,k}^{T}{\tilde{P}}_{k+1,k}^{-1}\Phi_{k+1,k}\right]}^{-1}\Phi_{k+1,k}^{T}{\tilde{P}}_{k+1,k}^{-1}\\ ={\tilde{P}}_{k+1,k}^{-1}+\Delta {\tilde{P}}_{k+1,k}^{-1} \end{array}$$

Then, the relation between $P_{k+1}$ and ${\tilde{P}}_{(k+1)i}$ can be given as

(41) $$\begin{array}{l} P_{k+1}={\left(H_{k+1}^{T}R_{k+1}^{-1}H_{k+1}+P_{k+1,k}^{-1}\right)}^{-1}\\ ={\left[{\tilde{H}}_{k+1}^{T}{\tilde{R}}_{k+1}^{-1}{\tilde{H}}_{k+1}+{\left(H_{k+1}^{i}\right)}^{T}\delta^{-2}H_{k+1}^{i}+{\tilde{P}}_{k+1,k}^{-1}+\Delta {\tilde{P}}_{k+1,k}^{-1}\right]}^{-1}\\ ={\left[{\tilde{P}}_{(k+1)i}^{-1}+{\left(H_{k+1}^{i}\right)}^{T}\delta^{-2}H_{k+1}^{i}+\Delta {\tilde{P}}_{k+1,k}^{-1}\right]}^{-1} \end{array}$$

When the convergence of Kalman filter is established, we can get $P_{k+1}\approx P_{k}$, comparing Equations (35) and (41), it can be obtained that

(42) $${\left({\tilde{P}}_{ki}^{-1}+{\left(H_{k}^{i}\right)}^{T}\delta^{-2}H_{k}^{i}\right)}^{-1}={\left[{\tilde{P}}_{(k+1)i}^{-1}+{\left(H_{k+1}^{i}\right)}^{T}\delta^{-2}H_{k+1}^{i}+\Delta {\tilde{P}}_{k+1,k}^{-1}\right]}^{-1}$$

If the maneuver of the vehicle is not very large and it moves at a relatively low speed, we can assume that $H_{k+1}^{i}$ hardly changes compared with $H_{k}^{i}$, and $H_{k+1}^{i}\approx H_{k}^{i}$ can be obtained. Hence, it can be obtained from Equation (42) that

(43) $${\tilde{P}}_{ki}^{jj}<{\tilde{P}}_{(k+1)i}^{jj}$$

Equation (43) indicates that the state estimation error will accumulate over a period of time. The rate of accumulation will be variant for different locations of the faulty measurement. In GNSS/INS integration, the state vector X includes attitude, velocity, and position, and they have different units. In order to evaluate the influence of a satellite’s pseudorange measurement on the precision of state estimation, two concepts are introduced. One is the precision of positioning (POP), which is chosen to evaluate the filter precision, and the definition of POP is expressed as

(44) $$\mathrm{POP}=\sqrt{\sum_{j=7}^{9}P_{k}^{jj}}$$

where $P_{k}^{jj}$ is the jth diagonal element of covariance matrix $P_{k}$. Although POP just contains the covariance of positioning error, it can still reflect the state estimation precision to some degree. And the smaller the POP, the higher the precision of state estimation.

The other one is the differential precision of positioning (DPOP), which is chosen to evaluate the influence of a satellite’s pseudorange measurement on the precision of state estimation, and it is defined as

(45) $$\mathrm{DPOP}=\sqrt{\sum_{j=7}^{9}{\tilde{P}}_{ki}^{jj}}-\sqrt{\sum_{j=7}^{9}P_{k}^{jj}},i=1,2,\cdots n$$

The above equation expresses the DPOP of the ith satellite, which is the difference between the POP calculated with all the satellites’ measurements except that of the ith satellite and the POP calculated with all the satellites’ measurements. If the ith satellite has the largest DPOP, it means that this satellite’s measurement has the greatest influence on the precision of state estimation and isolating this satellite’s measurement will result in the largest reduction of the state estimation precision. For this situation, if the fault lasts for a long time, the state estimation precision will decrease with time, which will result in the increase of MDB and the reduction of the sensibility of fault detection. On the contrary, if the ith satellite has the smallest DPOP among the n satellites, isolating this satellite’s measurement will result in the smallest reduction of the state estimation precision. For this situation, the duration time will have little effect on the fault detection performance.

DPOP reflects the influence of one satellite’s measurement on the precision of state estimation for a certain geometry of visible satellites. When the geometry varies, the influence of one satellite’s measurement on the precision of state estimation will be different. To analyze the performance of fault detection when fault occurs on the same satellite’s measurement in different geometries, a concept called relative differential precision of positioning (RDPOP) is introduced, and it can be expressed as

(46) $$\mathrm{RDPOP}=\frac{\sqrt{\sum_{j=7}^{9}{\tilde{P}}_{ki}^{jj}}-\sqrt{\sum_{j=7}^{9}P_{k}^{jj}}}{\sqrt{\sum_{j=7}^{9}P_{k}^{jj}}},i=1,2,\cdots n$$

It can be seen from the Equation (46) that RDPOP reflects the degree of the decrease of filtering precision after isolating the faulty measurement. For a given geometry, RDPOP has the same meaning as DPOP. When the geometry varies, RDPOP can be used to reflect the influence of the same satellite’s measurement on the filtering precision of different geometries. The larger the RDPOP, the greater the influence of the same satellite’s measurement on the filtering precision. When the number of satellites is the same, it can be concluded that if the RDPOP of the ith geometry is the largest among the n geometries, the fault detection performance in this geometry will be the worst for the same fault model occurring on the same satellite of the n geometries.

Besides, when the value of the fault is near the MDB, the probability of missed detection is still a little large. If the fault duration time is long, a large number of missed detection phenomena will happen, which will result in the decrease of state estimation precision. According to Equation (10), it can be seen that the fault detection function is related to the residual vector ${\bar{v}}_{k}$ and its covariance matrix $P_{\bar{v}k}$. When missed detection occurs, the component caused by error tracking will decrease the value of the test statistics and eventually result in the reduction of the sensibility of fault detection. As mentioned before, DPOP reflects the contribution of one satellite’s measurement to the precision of state estimation, the larger DPOP shows the greater contribution to the state estimation. Similarly, for faults with the same value, it can be obtained that the missed detection phenomena occurring on the satellite which has the larger DPOP will bring worse precision of state estimation, which will cause worse sensibility of fault detection. Therefore, for a given geometry of visible satellites, the same fault error occurring on different satellite’s measurement will lead to different results of fault detection.

## 5. Simulation Analysis

In this section, several numerical simulation tests are conducted to verify the above analysis. The influence of satellite configuration and fault duration time on the performance of fault detection is investigated. The simulation conditions are as follows:

The gyro constant drift is 0.1 °/h with its random drift 0.1 °/h, the constant bias and random bias of accelerometer is 50 μg. The initial geographical position is 108° east longitude, 34° north latitude, the initial velocity is zero, and the initial azimuth is 90°. The flight trajectory includes acceleration, deceleration, climbing motion, diving motion, and turning motion. The simulation time of flight trajectory is 1600 s. The standard deviation of GNSS pseudorange measurement error is 10 m. The false alarm rate and missed detection rate of fault detection are 0.001 and 0.2, respectively.

To verify the analysis, numerical simulation tests under different measurement conditions are conducted. The geometry of visible satellites is chosen randomly according to the real BDS geometry at Xi’an, China. The BDS simulation data is provided by a GNS8330 simulator. The output frequency of IMU and GNSS is 100 Hz and 1 Hz, respectively, and the Kalman filter cycle is 1 s.

The probabilities of missed detection and false alarm are commonly used to measure the performance of fault detection. Define alternative hypotheses $h_{i}(i=1,2,\cdots ,n)$, $h_{i}$ denotes that fault error occurs on the ith dimensional observation. Then, the probabilities of missed detection can be expressed as

(47) $$P_{{\mathrm{MD}}_{i}}=P\left\{{\bar{\Lambda }}_{k}<T_{d}/h_{i}\right\}$$

Usually, the probability of false alarm is given as

(48) $$P_{\mathrm{FA}}=P\left\{{\bar{\Lambda }}_{k}>T_{d}/H_{0}\right\}$$

In practical application, faults occurring at different dimensions of the measurement vector may result in different false alarm phenomena. Therefore, in this paper, we define a new probability of false alarm $P_{{\mathrm{FA}}_{i}}$, and it denotes the probability of a false alarm under the condition that fault error occurs on the ith dimensional observation. In the later tests, these two indices will be used to evaluate the performance of fault detection. In each test, a 10,000-run Monte Carlo simulation is conducted to obtain the statistical results of fault detection.

### 5.1. The Verification of the Influence of Satellite Configuration on Fault Detection for a Single-Epoch Fault

The influence of satellite configuration on the performance of fault detection consists of the geometry and number of visible satellites. Hence, the verification and analysis will be conducted from these two aspects respectively.

#### 5.1.1. Test on the Influence of the Geometry on Fault Detection

**Figure 1:**
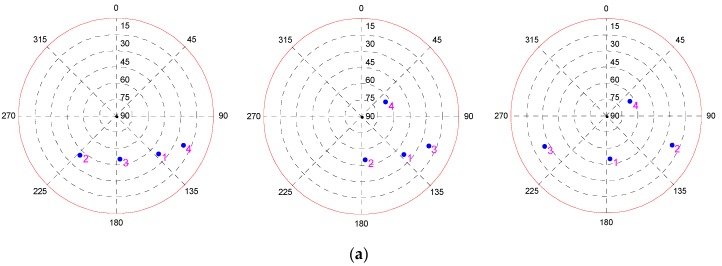

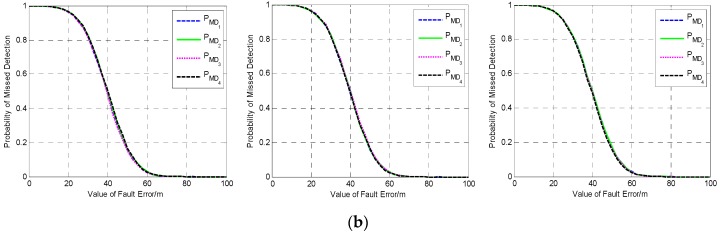
The fault detection results under three different geometries of four visible satellites. (**a**) Sky plot views of the satellite geometries; (**b**) The probabilities of missed detection under different alternative hypotheses as a function of fault error.

**Figure 2:**
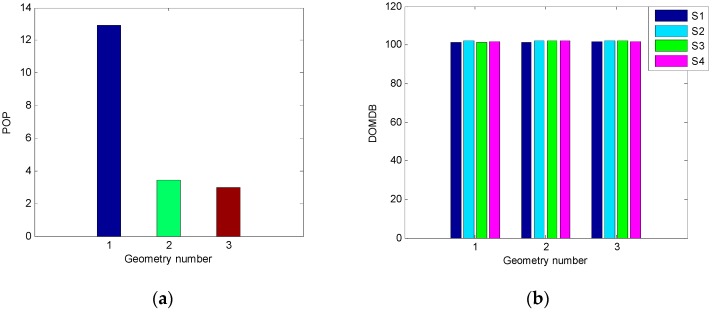
The comparison of precision of positioning (POP) and dilution of minimal detectable bias (DOMDB) of three different geometries. (**a**) POP; (**b**) DOMDB.

In this test, in order to analyze the influence of the geometry on fault detection, the performance of fault detection under several different geometries with four visible satellites are investigated. First, the fault detection results under three geometries in which the filtering error is not very large are investigated and shown in Figure 1. It can be seen that although the geometries of visible satellites are different, the fault detection results are nearly the same, and the MDB of each satellite in three geometries is about 48.5 m. In order to analyze the reason why this phenomenon happens, the POP and DOMDB of three geometries are calculated and the results are shown in Figure 2.

It can be seen from Figure 2a that the first geometry has the worst positioning precision among the three geometries and the precision is much lower than those of the other two geometries, while the third one has the best precision and the precision is a little higher than the second one. The results in Figure 2b indicate that the DOMDB of each satellite under three geometries are almost the same, the reason is that DOMDB mainly depends on the variance of measurement noise when the filter is converged. The similar DOMDB means that each satellite has almost the same MDB; therefore, the fault detection result for each satellite has little difference.

**Figure 3:**
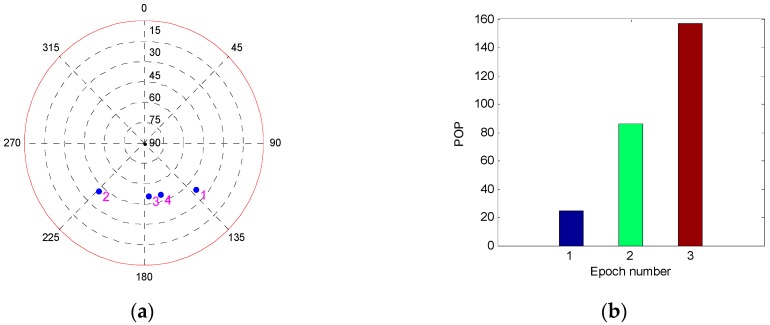

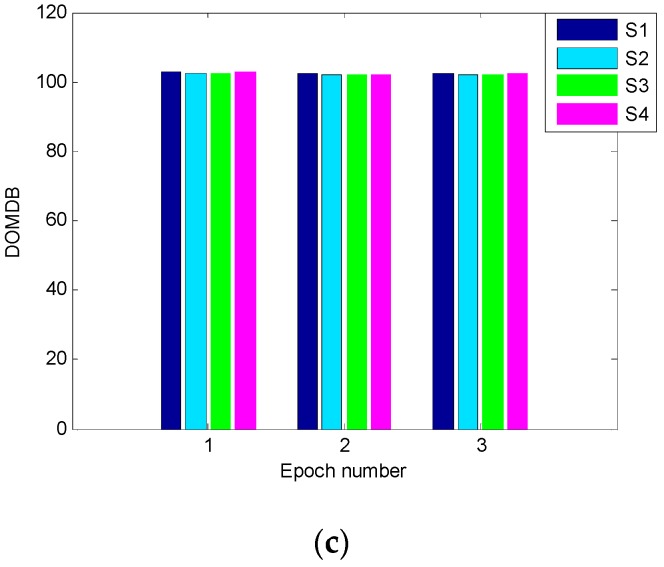
The comparison of POP and DOMDB of three different epochs under the new satellite geometry. (**a**) Sky plot view of the new satellite geometry; (**b**) POP; (**c**) DOMDB.

**Figure 4:**
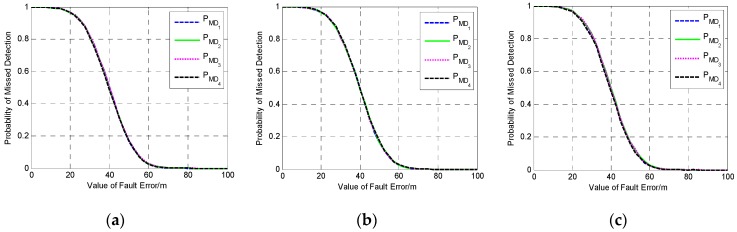
The fault detection results at three different epochs. (**a**) The detection results at the first epoch; (**b**) The detection results at the second epoch; (**c**) The detection results at the third epoch.

The above tests are based on the three geometries in which the Kalman filter is converged and the filtering error is not very large. To fully verify the influence of the geometry on the performance of single-epoch fault detection, a poor geometry in which the filtering error has a trend of divergence is selected to conduct a new simulation test. The fault detection performance at three different epochs with different filtering error is investigated and compared. The comparison of the POP and DOMDB under the new satellite geometry are shown in Figure 3, while the corresponding fault detection results are shown in Figure 4.

It can be seen from Figure 3a that the difference between the POP of the three epochs is noticeable, and the filtering error of the third epoch is very large. However, the result in Figure 3b indicates that the difference between the DOMDB of them is very small, which means that the sensibility of fault detection at these three epochs is nearly the same. Furthermore, the fault detection results in Figure 4 show that although the filtering error is very large and has large difference, but the fault detection result for a single-epoch is almost impervious. Comparing the fault detection results in Figure 1 and Figure 4, it can be obtained that the different satellite geometry will result in different filtering precision, but the fault detection result for single-epoch fault is less affected by the geometry.

#### 5.1.2. Test on the Influence of the Number of Visible Satellites on Fault Detection 

**Figure 5:**
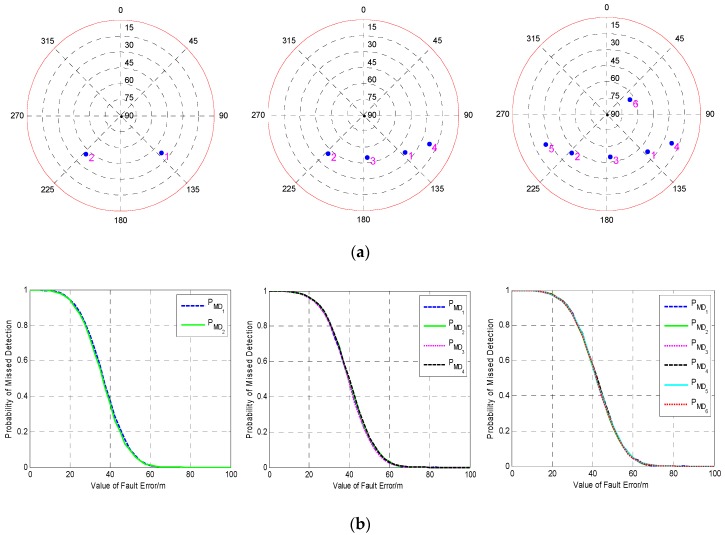
The fault detection results under two, four, and six visible satellites. (**a**) Sky plot views of the satellite geometries; (**b**) The probabilities of missed detection under different alternative hypotheses as a function of fault error.

In this test, in order to analyze the influence of the number of visible satellites on fault detection, the performance of fault detection under two, four, and six visible satellites is investigated, and the results are shown in Figure 5.

**Figure 6:**
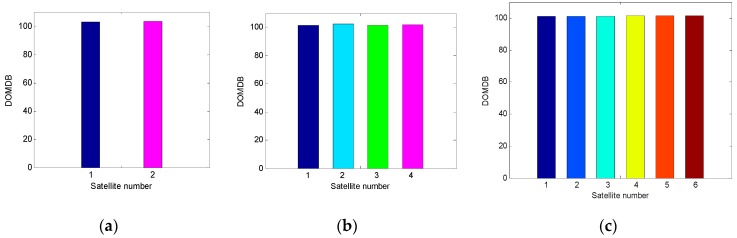
Comparison of each satellite’s DOMDB in the integration with two, four, and six visible satellites, respectively. (**a**) The results under two visible satellites; (**b**) The results under four visible satellites; (**c**) The results under six visible satellites.

It can be seen from Figure 5 that for faults with the same value, the probability of missed detection under two visible satellite is the lowest among the three cases, and the probability of missed detection increases with the number of satellites when the value of fault is between 20 m and 80 m. To analyze why this phenomenon happens, the DOMDB of each satellite in the integration with two, four, and six visible satellites is calculated respectively, and the results are shown in Figure 6.

It can be seen that the DOMDB of each satellite is nearly the same when the number of satellites is constant. When the number of satellites varies, the DOMDB changes a little, and the DOMDB under two visible satellites is a little larger than that under four and six satellites, this is because the filtering precision under two visible satellites is much lower than that under four and six satellites. Although the DOMDB mainly depends on the measurement noise covariance, when the filtering precision is very low, it will also have a great influence on the DOMDB. According to the analysis in Section 4.1, the MDB is related to both the noncentrality parameter σ and the DOMDB. When the number of satellites increases, the noncentrality parameter σ gets larger rapidly, while the DOMDB just changes a little, so the MDB increases with the number of satellites.

### 5.2. The Verification of the Influence of Satellite Configuration and Fault Duration Time on Fault Detection for a Continuous Fault

From the above tests in Section 5.1, it can be seen that both the geometry and number of visible satellites will affect the performance of fault detection for single-epoch fault, and the number of visible satellites has greater influence. Hence, in this section, the influence of fault duration time on fault detection under different number of visible satellites will be investigated by three tests firstly. The number of visible satellites is chosen as two, four, and six, respectively, and the geometries of them are the same as those in Section 5.1. Then, the influence of geometry on fault detection will be verified under three geometries with four visible satellites.

#### 5.2.1. Test on the Influence of the Fault Duration Time on Fault Detection with Two Visible Satellites

**Figure 7:**
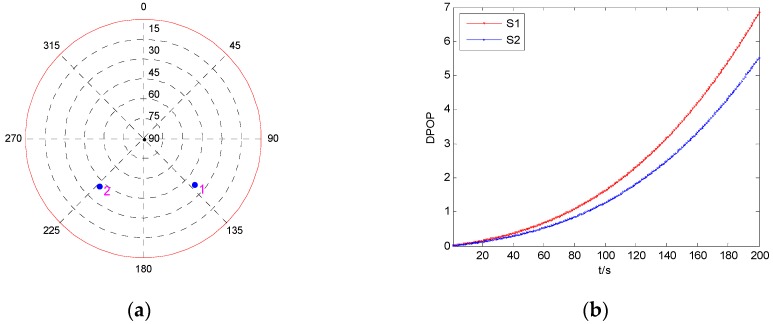
The geometry and DPOP of two visible satellites. (**a**) Sky plot view of the satellite geometry; (**b**) DPOP of each satellite as function of time.

For a single-epoch fault, the fault error occurring on a different satellite will hardly affect the performance of fault detection. However, isolating a different satellite’s measurement would result in the difference of state estimation precision, which may have variant influence on the later fault detection. Therefore, the DPOP of each satellite is investigated firstly to express the different influence of each satellite’s measurement on the state estimation precision, and the result is shown in Figure 7. The two satellites are marked as S1 and S2, respectively.

It can be seen from Figure 7 that both the DPOP of S1 and S2 have a tendency of rapid increase with time, and the DPOP of S1 is a little larger than that of S2. In other words, regardless of whether the fault error occurs on S1 or S2, isolating the faulty measurement will result in a great decline of positioning precision, and isolating the measurement of S1 will cause worse precision than isolating the measurement of S2.

**Figure 8:**
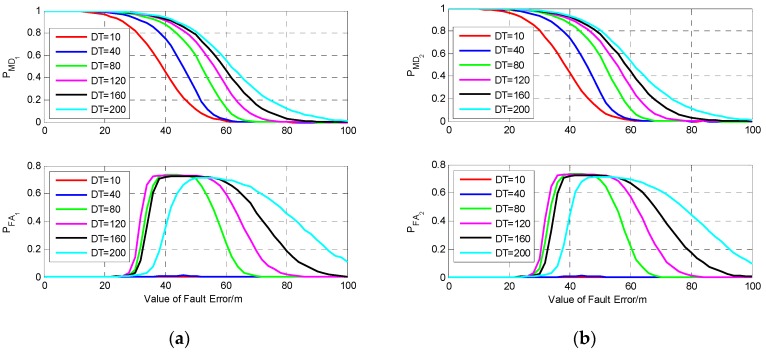
The results of fault detection with two visible satellites. (**a**) Fault occurs on S1; (**b**) Fault occurs on S2.

To verify the influence of fault duration time on detection performance, we set six different fault models, and the fault duration time of them is 10 s, 40 s, 80 s, 120 s, 160 s, and 200 s, respectively. The results of fault detection for fault error occurring on S1 and S2 are shown in Figure 8. From Figure 8, it can be seen that compared with the single-epoch fault, the fault detection performance for the continuous fault has obvious difference, and the probability of missed detection increases with the duration time of fault. For a single-epoch fault with the value equal to 60 m, the probability of missed detection is about 0.01, while for the same size of fault which lasts for 200 s, the probabilities of missed detection and false alarm are about 0.55 and 0.7, respectively. This comparison indicates that fault duration time has a great influence on fault detection performance. As there are only two visible satellites, isolating one of them will result in a great decline of the filtering precision; thus, for the same fault model, the fault detection performance for a fault occurring on S1 or S2 is nearly the same.

#### 5.2.2. Test on the Influence of the Fault Duration Time on Fault Detection with Four Visible Satellites 

**Figure 9:**
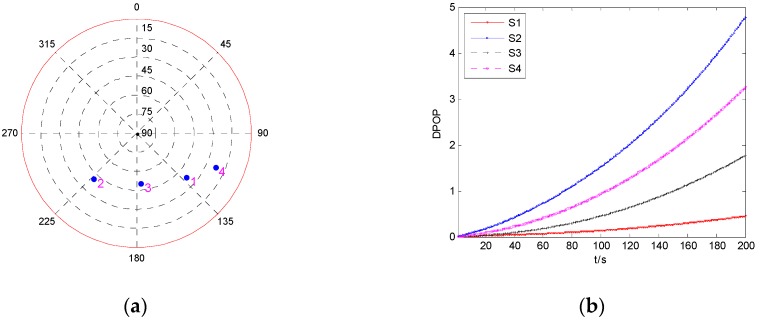
The geometry and DPOP of four visible satellites. (**a**) Sky plot view of the satellite geometry; (**b**) DPOP of each satellite as function of time.

Firstly, the DPOP of each satellite is investigated, and the result is shown in Figure 9. The four satellites are marked as S1, S2, S3, and S4, respectively.

**Figure 10:**
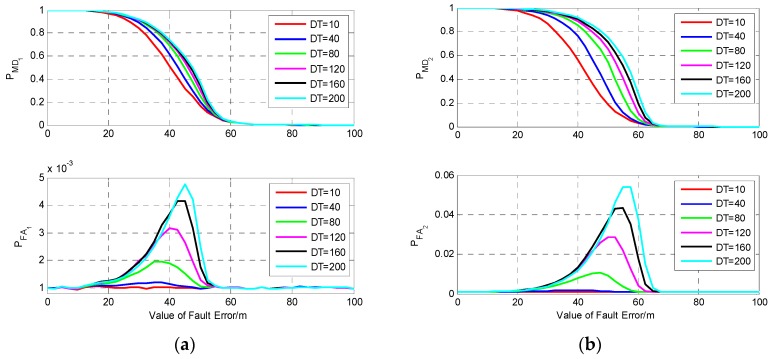
The results of fault detection with four visible satellites. (**a**) Fault occurs on S1; (**b**) Fault occurs on S2.

It can be seen from Figure 9b that among the four satellites, the largest DPOP happens when the measurement of S2 is isolated, while isolating S1 results in the minimum DPOP, and the tendency of the increase of DPOP is very slow. Hence, to analyze the influence of fault duration time on the performance of fault detection, the fault detection results for a fault error occurring on the measurements of S1 and S2 are investigated and shown in Figure 10. The fault models are the same as those in Section 5.2.1.

It can be seen from Figure 10 that the probability of missed detection increases with the increase of the fault duration time regardless of whether the fault occurs on S1 or S2. Comparing $P_{{\mathrm{MD}}_{1}}$ and $P_{{\mathrm{MD}}_{2}}$, it can be seen that the fault duration time has greater influence on $P_{{\mathrm{MD}}_{2}}$ than on $P_{{\mathrm{MD}}_{1}}$, and $P_{{\mathrm{MD}}_{2}}$ increases much faster than $P_{{\mathrm{MD}}_{1}}$ with the increase of fault duration time. For the same fault model, a fault occurring on S2 may cause larger probabilities of missed detection and false alarm than S1, especially when the value of fault is near the MDB. When the value of fault is large enough, the faulty measurement can be detected correctly without missed detection, and the probability of false alarm is nearly equal to the given value 0.001.

#### 5.2.3. Test on the Influence of the Fault Duration Time on Fault Detection with Six Visible Satellites

**Figure 11:**
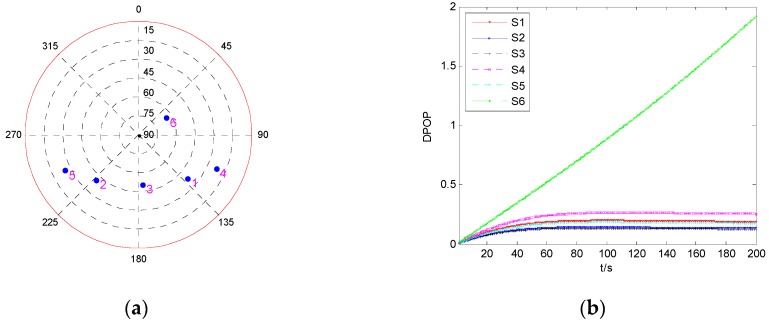
The geometry and DPOP of six visible satellites. (**a**) Sky plot view of the satellite geometry; (**b**) DPOP of each satellite as function of time.

Firstly, the DPOP of each satellite is investigated, and the result is shown in Figure 11. The six satellites are marked as S1, S2, S3, S4, S5, and S6, respectively.

**Figure 12:**
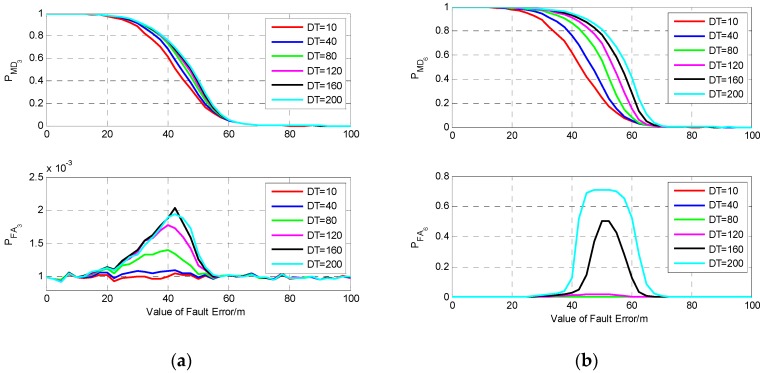
The results of fault detection with six visible satellites. (**a**) Fault occurs on S3; (**b**) Fault occurs on S6.

From Figure 11b, it can be seen that the DPOP of S6 has a faster trend of increase compared with those of the other five satellites, while the tendency of the other five DPOP is very slow, and among them, the DPOP of S3 is the smallest. This means that isolating the measurement of S6 will result in the largest decline of positioning precision, while when a fault occurs on one of the other five satellites, isolating the faulty measurement will cause relatively smaller decrease of precision, and isolating the measurement of S3 will bring the smallest decline. The fault detection results for fault error occurring on the measurements of S6 and S3 are investigated and shown in Figure 12.

It can be seen from Figure 12 that the fault duration time has greater influence on $P_{{\mathrm{MD}}_{6}}$ than $P_{{\mathrm{MD}}_{3}}$. For the same fault with small value, fault occurring on S6 may cause larger probability of missed detection and false alarm than S3. In Figure 12b, when the value and duration time of fault are 60 m and 200 s, respectively, $P_{{\mathrm{MD}}_{6}}$ is about 0.41, and $P_{{\mathrm{FA}}_{6}}$ is about 0.52, which means that a large number of missed detections and false alarms happen under this fault model, and the probability is much higher than that of single-epoch fault detection. While for the same fault model occurring on S3, it can be seen that $P_{{\mathrm{MD}}_{3}}$ and $P_{{\mathrm{FA}}_{3}}$ are about 0.05 and 0.001, which is nearly the same as that of single-epoch fault detection.

**Figure 13:**
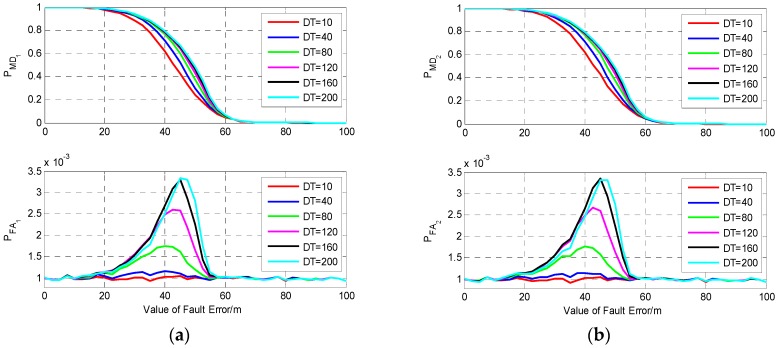
The results of fault detection with six visible satellites. (**a**) Fault occurs on S1; (**b**) Fault occurs on S2.

To verify the influence of the increase of visible satellites number on the performance of fault detection for the same satellite, the fault detection results with six visible satellites for a fault occurring on S1 and S2 are investigated and shown in Figure 13.

Comparing Figure 8, Figure 10, and Figure 13, it can be seen that for the same fault model, when the fault duration time is relatively short, the fault detection performance under two visible satellites is better than that under four and six visible satellites. But, when the fault duration time gets longer, the influence of fault duration time on the fault detection performance will decrease with the increase of the visible satellites number. When the fault duration time increases to a certain value, the greater the number of visible satellites, the better the fault detection performance. The reason is that the influence of one satellite’s measurement on the navigation precision will decrease with the increase of the number of visible satellites. This conclusion can also be obtained from the comparison of the DPOP of S1 and S2 in Figure 7, Figure 9, and Figure 11. Therefore, it can be seen that when the number of visible satellites is large enough, isolating the faulty measurements will not bring large degradation of filtering precision and fault detection performance.

#### 5.2.4. Test on the Influence of the Geometry on Fault Detection for Continuous Fault with Four Visible Satellites

**Figure 14:**
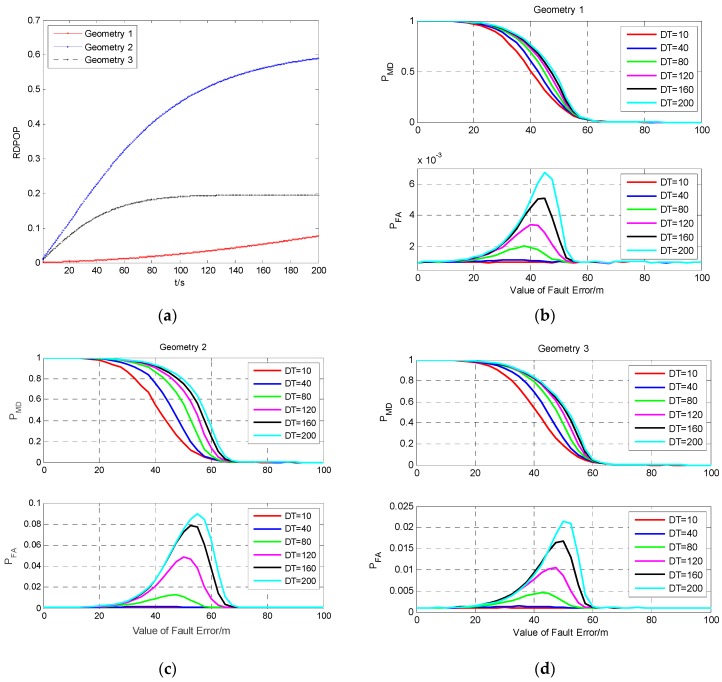
Comparison of the RDPOP of G1 and fault detection results in three geometries. (**a**) The RDPOP of G1 in three geometries; (**b**) Fault detection results in geometry 1; (**c**) Fault detection results in geometry 2; (**d**) Fault detection results in geometry 3.

To verify the influence of the geometry on the detection performance for a continuous fault, the fault detection results for a fault occurring on the same satellite of different geometries with four visible satellites are investigated. It can be seen from Figure 1 that S3 in geometry 1, S2 in geometry 2, and S1 in geometry 3 are the same satellite. Here, we mark this satellite as G1, and the fault detection results for fault occurring on G1 in three geometries are shown in Figure 14.

It can be seen from Figure 14a that the RDPOP in geometry 2 is the largest among the three cases, while that in geometry 1 is the smallest, which means that the satellite G1 has the greater influence on the filtering precision in geometry 2 than in geometries 1 and 3. Figure 14b–d shows that, for the same fault model, especially when the value of fault error is near the MDB, the probabilities of missed detection and false alarm in geometry 2 are higher than those in geometries 1 and 3. This conclusion is consistent with the result in Figure 14a.

## 6. Conclusions

In this paper, the principle and performance of the residual chi-square detection method, which is the commonly used method in the field of fault detection for GNSS/INS integration, are investigated. The equivalence deduction of chi-square detection based on the predicted residual and least-squares residual is given. Then, to examine the performance of residual chi-square detection under different observational conditions and fault models, the influence of some important factors, such as the number and geometry of visible satellites and the fault duration time, on the performance of fault detection is analyzed in theory. To verify the theoretical analysis, several numerical simulations are conducted. The main conclusions of simulation tests are listed as follows.

1. For single-epoch fault detection, when the number of satellites is given, the geometry of satellites has little effect on the performance of fault detection, and the MDB of each satellite is nearly the same. When the number of satellites varies, the MDB increases with the increase of satellite number. But, regardless of which satellite’s measurement has fault error, the difference of fault detection results will still be very small. Therefore, there is no need to adopt a different detection method for a fault occurring on a different satellite.

2. For continuous fault detection, the probability of missed detection increases with the increase of fault duration time when the value of the fault is within a certain range, especially near the MDB. For a given geometry, the fault duration time has greater influence on the satellite which has larger DPOP. When the number of satellites is given, the fault duration time has different influence on the same satellite in different geometries, and the larger the RDPOP, the greater the influence. When the number of satellites increases, the influence of fault duration time on the fault detection performance will decrease.

In practical application, the measurement conditions and fault models may be variant and unknown. For continuous fault detection, these factors will have great influence on the fault detection performance. Ignoring the influence of them and adopting the same fault detection and isolation method for different situations may result in bad fault detection and filtering precision. Therefore, it’s very important to adopt different fault detection and isolation methods for different measurement conditions and fault models. According to the research results of this work, future research work will focus on how to realize the adaptive adjustment of fault detection, isolation, and adaptation according to the number and geometry of visible satellites.
